# Examining the Relationship between Pre-Malignant Breast Lesions, Carcinogenesis and Tumor Evolution in the Mammary Epithelium Using an Agent-Based Model

**DOI:** 10.1371/journal.pone.0152298

**Published:** 2016-03-29

**Authors:** Joaquin Chapa, Gary An, Swati A. Kulkarni

**Affiliations:** 1 Pritzker School of Medicine, University of Chicago, 924 East 57th Street #104, Chicago, Illinois, 60637, United States of America; 2 Department of Surgery, University of Chicago, 5841 S. Maryland Ave, Chicago, Illinois, 60637, United States of America; 3 Department of Surgery, Northwestern University, Robert H. Lurie Comprehensive Cancer Center, 303 E Superior Street, Lurie, 4–105, Chicago, Illinois, 60611, United States of America; University of Wisconsin—Madison, UNITED STATES

## Abstract

**Introduction:**

Breast cancer, the product of numerous rare mutational events that occur over an extended time period, presents numerous challenges to investigators interested in studying the transformation from normal breast epithelium to malignancy using traditional laboratory methods, particularly with respect to characterizing transitional and pre-malignant states. Dynamic computational modeling can provide insight into these pathophysiological dynamics, and as such we use a previously validated agent-based computational model of the mammary epithelium (the DEABM) to investigate the probabilistic mechanisms by which normal populations of ductal cells could transform into states replicating features of both pre-malignant breast lesions and a diverse set of breast cancer subtypes.

**Methods:**

The DEABM consists of simulated cellular populations governed by algorithms based on accepted and previously published cellular mechanisms. Cells respond to hormones, undergo mitosis, apoptosis and cellular differentiation. Heritable mutations to 12 genes prominently implicated in breast cancer are acquired via a probabilistic mechanism. 3000 simulations of the 40-year period of menstrual cycling were run in wild-type (WT) and BRCA1-mutated groups. Simulations were analyzed by development of hyperplastic states, incidence of malignancy, hormone receptor and HER-2 status, frequency of mutation to particular genes, and whether mutations were early events in carcinogenesis.

**Results:**

Cancer incidence in WT (2.6%) and BRCA1-mutated (45.9%) populations closely matched published epidemiologic rates. Hormone receptor expression profiles in both WT and BRCA groups also closely matched epidemiologic data. Hyperplastic populations carried more mutations than normal populations and mutations were similar to early mutations found in ER+ tumors (telomerase, E-cadherin, TGFB, RUNX3, p < .01). ER- tumors carried significantly more mutations and carried more early mutations in BRCA1, c-MYC and genes associated with epithelial-mesenchymal transition.

**Conclusions:**

The DEABM generates diverse tumors that express tumor markers consistent with epidemiologic data. The DEABM also generates non-invasive, hyperplastic populations, analogous to atypia or ductal carcinoma *in situ* (DCIS), via mutations to genes known to be present in hyperplastic lesions and as early mutations in breast cancers. The results demonstrate that agent-based models are well-suited to studying tumor evolution through stages of carcinogenesis and have the potential to be used to develop prevention and treatment strategies.

## Introduction

### Heterogeneity and complexity in breast cancer

Breast cancer is a highly heterogeneous condition arising via innumerable different pathway alterations, that are themselves caused by progressive genetic insults [1, 2]. The expansion in knowledge of the genetic alterations underlying breast carcinogenesis has led to an increasing awareness of the heterogeneity in the entities that are aggregated under the label “breast cancer” [3]. Clinical decision making for invasive breast cancer is being increasingly informed by molecular properties of the tumor, including hormone receptor or HER2 overexpression), and Oncotype DX has made analysis of gene expression for both prognosis and predicting response to therapy clinically relevant [4, 5]. The attempt to classify breast cancer into molecular subtypes continues to be the subject of intense research, and has proven to be important in guiding treatment decisions and predicting prognosis [6, 7]. Our emerging understanding of breast cancer as a complex, heterogeneous collection of disease states presents formidable challenges to the traditional, reductionist research methods employed today [8]. Despite intensive effort and investment to identify frequently mutated genes, altered protein expression and dysregulated pathways, the answers to many fundamental questions about breast cancer biology remain elusive.

Among the most clinically relevant of these questions are those concerning the processes by which normal tissue transforms and acquires the behavioral hallmarks of cancer. In the breast epithelium a number of pathologic conditions have been identified, ranging from Usual Hyperplasia to Atypia to Ductal Carcinoma in Situ (DCIS), each of which confers an increased risk of developing invasive breast cancer [9–12]. To what extent these lesions represent a continuum of epithelial transformation from normal to frankly malignant remains a subject of considerable debate. [10, 13, 14]. An understanding of the processes by which these preneoplastic lesions arise, and how the genetic and downstream behavioral alterations cause premalignant lesions to transform and acquire the invasive, immortal phenotype that defines malignancy will be essential in answering these questions. A greater understanding of the processes by which breast cancers evolve from precursor lesions could potentially guide clinical and therapeutic decision making for patients with proliferative breast lesions who are at increased risk for developing invasive breast cancer.

Given the probabilistic and complex nature of cancer—it’s development from innumerable, interacting and rare genetic alterations—it seems likely that new methods will be required to supplement traditional *in vitro* and *in vivo* research models. Here we propose agent-based modeling—in which computational agents are programmed to execute algorithmic behavior programs based on known cellular and molecular mechanisms—as a research methodology to model and study the biology of the mammary ductal epithelium, and as a tool to examine the controversial issues surrounding proliferative states that are difficult to study via traditional methods.

### Computational modeling and breast cancer research

Agent-based models (ABMs) offer a useful and intuitive way to employ the knowledge created by traditional *in vitro* and *in vivo* experiments to create a functional map of biological systems as they are currently understood [15–17]. In an ABM of a multi-cellular system, known biological mechanisms are encoded into algorithms that govern the behaviors of *agents*—computational entities that abstractly represent biological cells. ABMs can be used to create dynamic simulations in which virtual cells continually interact both with each other and their environment in a concurrent and parallel fashion, characteristics that make ABMs favorable to studying complex cellular systems. Because cells can be programmed to continually record variables that describe their current state and behavior, and because those variables are then observable by the experimenter, ABMs allow for data and information extraction at a level of resolution and detail not possible in either more traditional experimental or clinical research environments. Using these principles of ABMs, in previously published work we demonstrated that our Duct Epithelium Agent-Based Model (DEABM) was able to create a population consisting of luminal epithelial cells, myoepithelial cells and fibroblasts that reproduced the stable cell-cell signaling dynamics of the mammary epithelium in response to physiologically cycling estrogen and progesterone[18]. After implementing an algorithm to produce genetic mutations via probabilistic DNA damage and repair mechanisms, the DEABM generated simulated tumors matching age-specific incidences similar to published epidemiologic data.

Traditional *in vivo* and *in vitro* breast cancer research tends to either focus on one particular aspect of tumor biology, such as on how alterations in single genes or proteins affect a phenotype (e.g. response to potential therapeutics), or on cross-sectional gene expression profiling that represents a snapshot of a tumor at a single point in time. ABMs, by allowing agents to interact with each other and undergo random, sequential mutations to genes that affect functional behaviors, integrates dynamic, mechanism-driven processes (i.e. how gene mutations alter individual cellular behaviors) with tumor-level characteristics (i.e. cell-population characteristics) to provide greater contextual representation and understanding of the complex and dynamic pathogenesis of breast cancer. The DEABM, because of its stochastic process for generating mutations and inheritance of genetic mutations passed from parent to daughter cells, is able both to demonstrate evolutionary dynamics of tumor development and generate heterogeneous populations of cells that can transform into tumors with varying genetic and functional characteristics. These unique properties of ABMs have made it a popular mechanism of inquiry and an important adjunct to more traditional research methods in a diverse array of biological contexts, including cellular architecture in DCIS [19], the immune response in sepsis [20], wound healing [21], and necrotizing enterocolitis [22], as well as in other fields facing similar problems with complex systems such as economics [23], ecology [24] and network theory[25].

### The DEABM: exploring challenging questions in breast cancer

Recognizing the importance and extent of heterogeneity in breast cancer, and with the goal of exploring how ABMs can improve our risk stratification of pre-malignant breast lesions, we expanded the DEABM to further account for pathways known to be important in the pathogenesis of breast cancer subtypes. The previous version of the DEABM could generate only ER+ or ER- tumors, and development of cancer was the only outcome examined. This updated version of the DEABM is capable of generating tumors expressing any combination of ER or HER2 status, and tumors can be analyzed for mutations in any of the genes in the DEABM’s functional genome, as well as the sequence in which mutations occurred. We use this updated model to examine the progression of mutations and their functional consequences in the progression from normal mammary tissue dynamics to malignancy.

In particular, pre-malignant lesions are of concern because they represent an area where intervention—preventing the progression to invasive breast cancer—can have far- reaching downstream effects. At present, clinical decision making is based largely on pathologic diagnoses rather than markers reflecting the underlying biology and there is significant controversy about how to assess the risk of pre-malignant lesions and what interventions, if any, should be offered to patients with these lesions [10, 13, 14]. Much of this ambiguity stems from our incomplete understanding of the origins, progression and natural history of breast cancer as the endpoint of the long, probabilistic and complex process of oncogenesis.

While much remains unknown about how tissues undergo the changes required for malignant transformation, recent studies have indicated that as many as half of the mutations present in malignancies may originate prior to the onset of neoplasia [26]. Correspondingly, studies indicate that proliferative lesions [27] and DCIS [28] more frequently harbor mutations in genes known to be mutated in invasive breast cancer. Many of these genes are incorporated into the functional genome of the DEABM, including p53 [29, 30], c-Met [31], genes controlling the PI3K pathway [32], ER-alpha [33], E-cadherin [34] and c-MYC[34]. A greater understanding of the pathologic processes leading to premalignant lesions is required to fully understand them, and to assess their risk for progression [35]. Here we use the DEABM to examine the specific case of breast tissue by modeling the progression from normal epithelial dynamics to malignancy, with a particular emphasis on how genetic alterations differ between normal, hyperplastic and malignant lesions.

## Methods

### Overview of the DEABM

The DEABM primarily focuses on how genetic mutations alter cellular behaviors: their effects on cell-cell signaling pathways regulating proliferation, response to estrogen, apoptosis and other features central to how malignant cell populations differ from normal populations. Because it is not feasible to model every molecular process in a population of cells, the DEABM treats other features of mammary gland biology abstractly. For example, the DEABM does not seek to model the complex three-dimensional architecture of the mammary duct system and how the physical mechanics of cell-cell interactions play out in the normal versus cancerous state.

**Fig 1:**
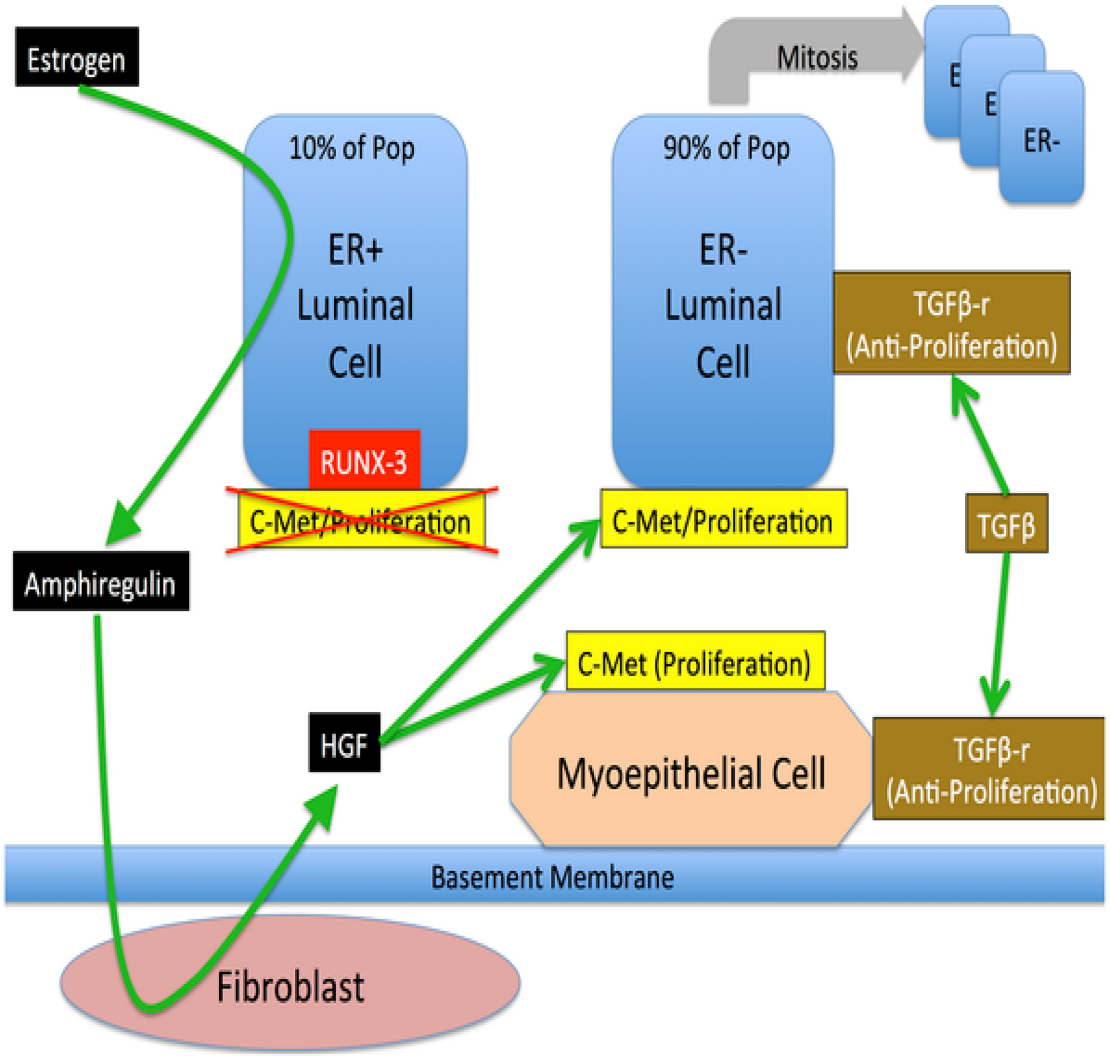
Overview of paracrine signaling involved in maintenance of the breast ductal epithelium in the Duct Epithelium Agent-based Model (DEABM). In normal breast tissue ER+ luminal cells respond to estrogen by producing amphiregulin, which stimulates production of the mitogen Hepatocyte growth factor (HGF) by fibroblasts. HGF in turn stimulates proliferation in ER- luminal cells and myoepithelial cells by binding to C-Met. Figure reproduced from [18] under the Creative Commons Attribution license.

**Fig 2:**
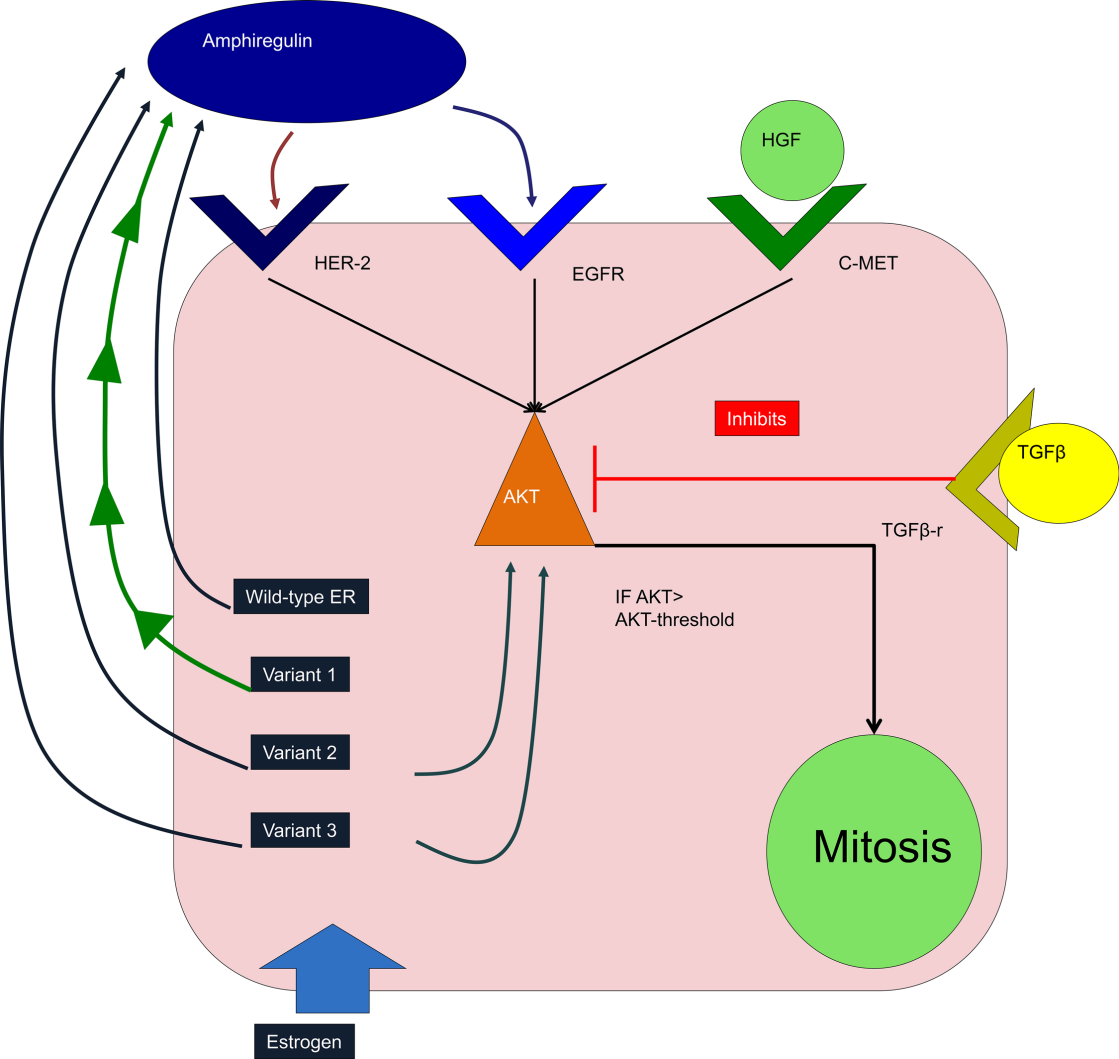
Overview of intracellular signaling for duct epithelial cells in the Duct Epithelium Agent-based Model (DEABM). Estrogen initiates the production of amphiregulin in the Wild-type ER + condition. In turn amphiregulin, along with HGF produced by fibroblasts, increases intracellular levels of AKT; AKT levels rising beyond a set threshold trigger mitosis. Transforming growth factor beta (TGF-β) inhibits growth by decreasing intracellular AKT. The DEABM allows for mutations resulting in hypothesized variant forms of ER that have varying growth promoting effects: Variant 1 causes cells to autonomously produce high levels of amphiregulin. Variant 2 causes estrogen to directly increase intracellular AKT, mimicking a form of non-genomic ER signaling. Variant 3 is a combination of Variants 1 and 2, causing cells to produce a moderate amount of autonomous amphiregulin as well as a moderate amount of direct AKT increase.

The DEABM represents the ductal epithelium two-dimensionally, as a layer of fibroblasts overlaid by myoepithelial cells, both of which are overlaid by luminal epithelial cells. Cell variables describe internal molecular components of the cell that include DNA integrity, genetic mutations present, expression of hormone or membrane receptors, telomere length and internal cell signaling molecules. Cellular agents execute their rule sets as the simulation iterates, thus interacting with other agents as well as the environment. The paracrine and intracellular mechanisms of the cell-cell interactions, as represented by the DEABM, are depicted in Figs 1 and 2.

The DEABM simulates time in step-wise intervals, with each step representing one day of simulated time. At the end of each day, cells execute their behavioral algorithms, environmental variables are updated, and estrogen and progesterone levels in the environment update according to a 28-day menstrual cycle. The DEABM allows for simulations of long periods of time—40 year periods in the current study—which allows a focus on the longitudinal, probabilistic nature of cancer development over years of DNA stress/repair cycles and potential errors in mitosis. This addresses an important challenge in studying complex disease processes like cancer: to effectively characterize the pathophysiology of a disease process like cancer requires that a model be able to characterize the transition from the functioning, healthy, baseline state to the altered, pathologic state.

### The Functional Genome of the DEABM

The cellular agents in the DEABM incorporate eleven genes that govern functions known to be both critical in the dynamics of the normal epithelium, as well as known to be frequently altered in breast cancers. Genes regulate critical biologic behaviors such as DNA damage repair (BRCA1, P53), cellular proliferation and response to hormones and growth factors (HER2, TGF-beta, EGFR, RUNX3, c-MYC, ESR-1), apoptosis and cellular senescence (Telomerase, P53), cell-cell adhesion (E-cadherin) and invasion beyond the basement membrane (Matrix Metalloprotease 3).

**Fig 3:**
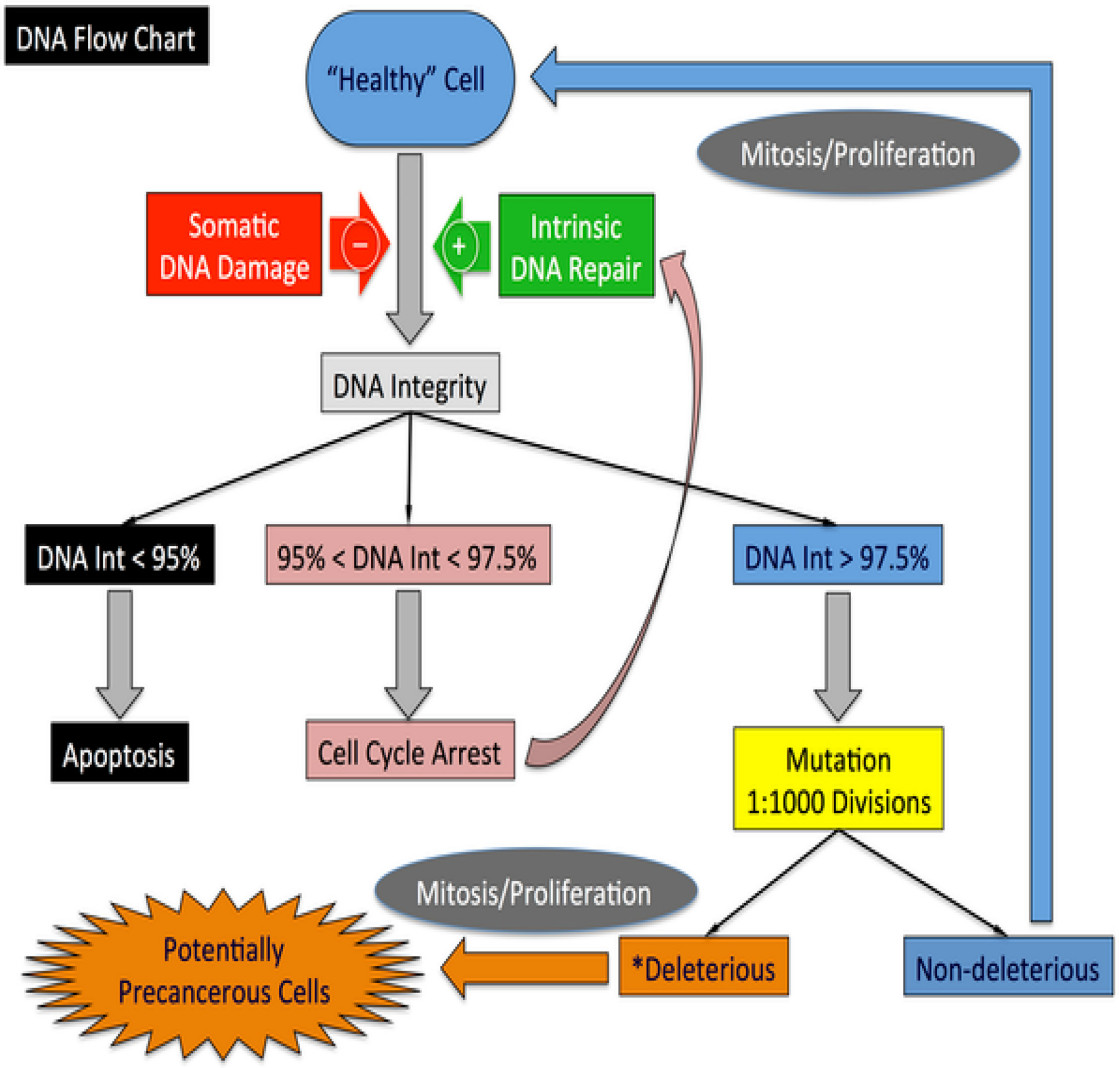
Overview of mechanisms governing DNA damage and repair and mutation in the Duct Epithelium Agent-based Model (DEABM). Cells acquire and repair a random but limited amount of DNA damage every day. Cells dividing with DNA damage can acquire mutations. Excessive amounts of DNA damage lead to either cellular senescence or apoptosis. If damage occurs to one of the copies of the specifically labeled “functional genes” there is no functional effect. If both copies of a gene is mutated the respective functions associated with that genes are modified, leading to altered cellular behaviors. Figure reproduced from[18] under the Creative Commons Attribution license.

HER-2 is a new addition to the DEABM’s functional genome, and was added as it is both a driver of proliferation in a minority of breast cancers and also represents a clinically important breast cancer subtype [36]. By default, DEABM luminal cells in the normal state do not express HER-2. Mutations to HER-2 can lead to a suppression of inhibition of HER-2 and augmentation of the proliferative response to mitogens. Additionally, three variant mutations affecting the estrogen receptor, reflecting previously described mutations found in ER+ breast cancers, are now possible in the DEABM. These mutations affecting ESR1 allow for variant behavior of ER and have various growth-promoting effects. The effect of including these variants is to increase the diversity of behavioral profiles of nominally ER+ tumors to more closely reflect the diversity present in ER+ tumors observed clinically. Variant 1 causes autonomous production of the growth factor amphiregulin, whose function is detailed in Fig 1 and explained in the Supplemental Methods (S1 Text), regardless of the supply of estrogen available to ER+ cells. Variant 2 causes a non-genomic, direct promotion of proliferation effect via increased intracellular AKT, representing a known mechanism of non-genomic, pro-proliferation activation of receptor tyrosine kinases by ER in certain breast cancers[37]. Variant three combines the effects of variants 1 and 2, with both autonomous growth factor production and direct stimulation of proliferation. These mutation effects could represent mutations to ESR1 itself [33], epigenetic alterations to ER function[38], or splice site alterations causing variant ER function[39]. The list of genes modeled by the DEABM and their associated functional effects are illustrated in Fig 3.

Cells possess two copies of each gene in the normal state. During each simulated day within the model, cells acquire and repair a random but limited amount of DNA damage, which represent the oxidative and environmental insults faced by cells *in vivo*. Additionally, cells undergoing mitosis have a probability of making replication errors. Cells that divide with accumulated unrepaired DNA damage face a probability of acquiring a mutation to a functional gene; the probability of a deleterious mutation occurring is directly related to the amount of DNA damage acquired prior to division. Cells must acquire two mutations to a given gene before gain or loss of function effects are manifested in cellular behavior.

**Fig 4:**
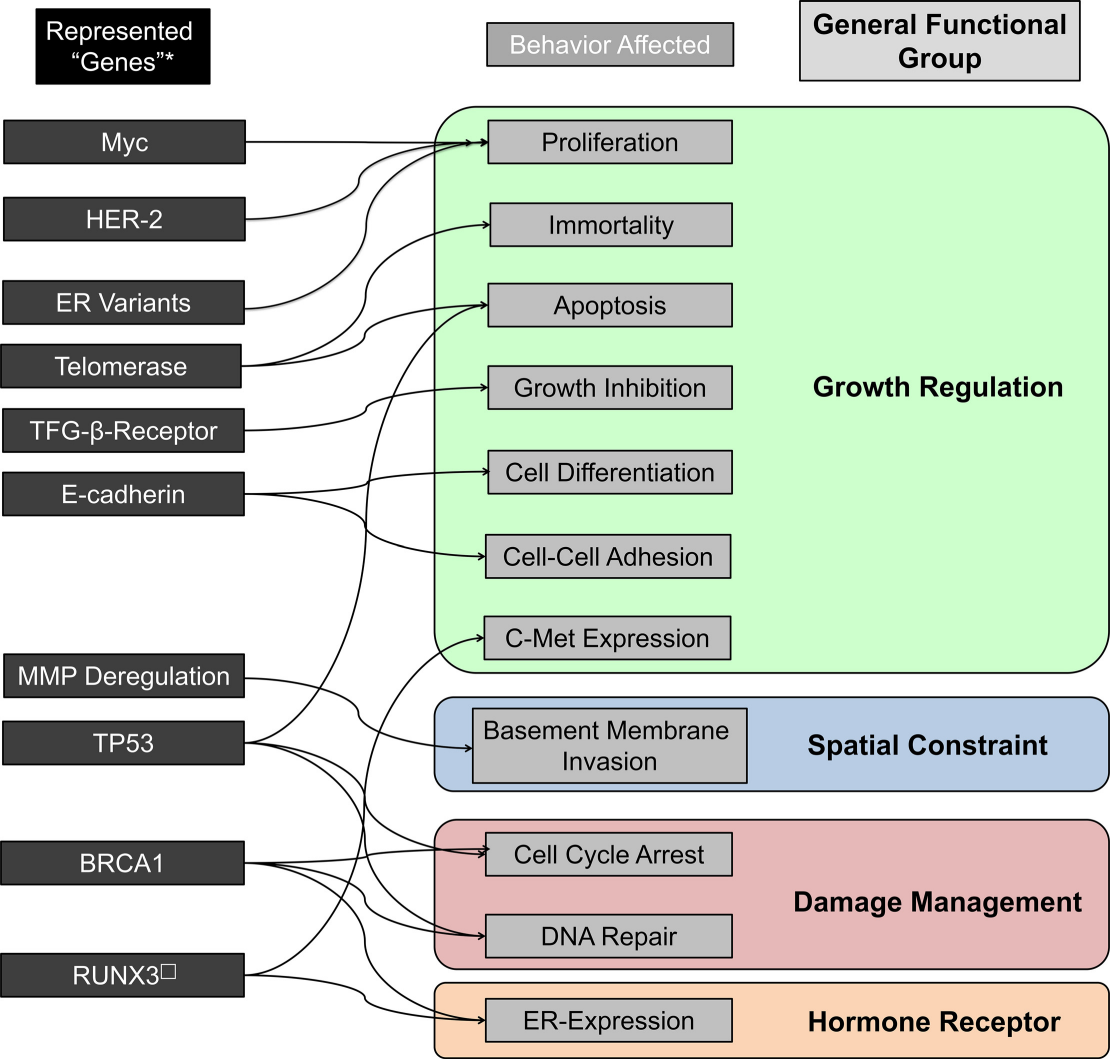
Overview of gene-function relationships in the Duct Epithelium Agent-based Model (DEABM). Mutations to individual genes can have single or multiple effects on cellular behavior. Gene-function categories in the DEABM can be generally grouped into the categories of growth regulation, spatial constraint, damage management and hormone receptor function. In order for cells in the DEABM to acquire the behavioral hallmarks of malignancies, multiple mutations in multiple categories must be acquired.

Each gene within the DEABM is mapped to particular cellular behaviors as dictated by the algorithms the DEABM comprises. Loss of a gene’s function thus leads to altered cellular behavior. Loss of DNA damage repair proteins leads to reduced ability to repair damaged DNA, with resulting increased probability of further mutations (see Fig 4). Unregulated growth factor receptors have proliferation promoting effects. Loss of a tumor suppressor causes failure to suppress proliferation at cell-cycle checkpoints. Loss of cell-cell adhesion proteins causes impaired apoptosis of unadhered cells. Full descriptions of the specific effects of mutations on the behavior of cells in the DEABM can be found in the supplementary methods (S1 Text).

Mutations in the DEABM are heritable: daughter cells contain the same genome as the parent cells of which they are a product. As mutated cells continue to acquire functional mutations and pass them down via mitosis, it is possible for lineages of cells to exhibit increasingly altered behavior. No single mutation is sufficient to cause the malignant phenotype within the DEABM; combinations of multiple mutations, involving different aspects of cellular behaviors, are required to cause the uncontrolled proliferation, impaired apoptosis and invasive behavior required for the development of malignancy.

### Simulation Experiments

DEABM simulations were performed to represent a population of individual virtual patients’ breast tissue over 40-years, reflecting the average time between menarche and menopause. Full details of simulation parameters and cells’ behavior algorithms can be found in the supplemental methods (S1 Text).

Simulations were allowed to run for either 40 years or until the development of malignancy, with malignancy defined as an expansion of the luminal cellular population by greater than 10-fold and invasion beyond the basement membrane. At the conclusion of the simulation, cell populations were characterized as malignant, normal or hyperplastic. Hyperplastic lesions were identified at the end of the simulated period, and defined as cell populations with greater than 2x the cellular population of luminal cells in the normal state. Because the clinical characterization of premalignant breast lesions is a pathologic rather than molecular or genetic diagnosis, the DEABM does not represent features such as nuclear atypia and cytologic architecture used to distinguish lesions. We did not separate hyperplastic lesions into pathologic categories (e.g. usual hyperplasia, atypia and DCIS) and they are treated as a group.

Three thousand simulations were run in each of two groups: a wild-type group that contained two functioning copies of each of the twelve genes in the DEABM’s functional genome, and a BRCA-1 mutated group, in which cells began the simulation with only a single copy of BRCA-1, making them more susceptible to a “second hit” that led to increased genomic instability. The ability of the DEABM to faithfully model both a normal and a BRCA1 mutated state allows the DEABM to explore how inherited mutations in breast cancer might lead to both increased susceptibility to cancer, as well as the tendency for BRCA1-associated cancers to develop as hormone-receptor negative subtypes. Outcome measures were chosen to determine whether malignancies generated by the DEABM matched known data on breast cancers, and included cumulative cancer incidence over time, ER and HER-2 expression, sequential ordering of mutations acquired, and total number of mutations to functional genes. Cells were considered “ER+” if greater than 9% of cells expressed ER, a value that corresponds to physiologic expression of ER in normal breast tissue and the baseline rate of ER expression in the DEABM [40–43]. Tumors were considered HER-2+ if greater than 5% of cells expressed HER-2. In the model, PR expression is dependent on ER expression and therefore not explicitly reported in our results [44]. Based on ER and HER-2 expression, we have grouped our tumors into four subtypes that roughly approximate the four commonly utilized molecular subtypes: ER+/HER- (Luminal A); ER+/HER+ (Luminal B); ER-/HER+ (HER-2 positive) and ER-/HER-2- (basal). In analyzing the sequential order of mutations, mutations were defined as “early” if they were one of the first 5 mutations acquired by a cell lineage. Cancer incidence and tumor subtype incidence were compared to previously published epidemiological rates. Mutation frequency of individual genes was compared between malignancies, hyperplastic, and normal cell populations generated by the DEABM simulations.

## Results

### Cancer incidence and tumor subtype: DEABM vs. Epidemiologic data

**Fig 5:**
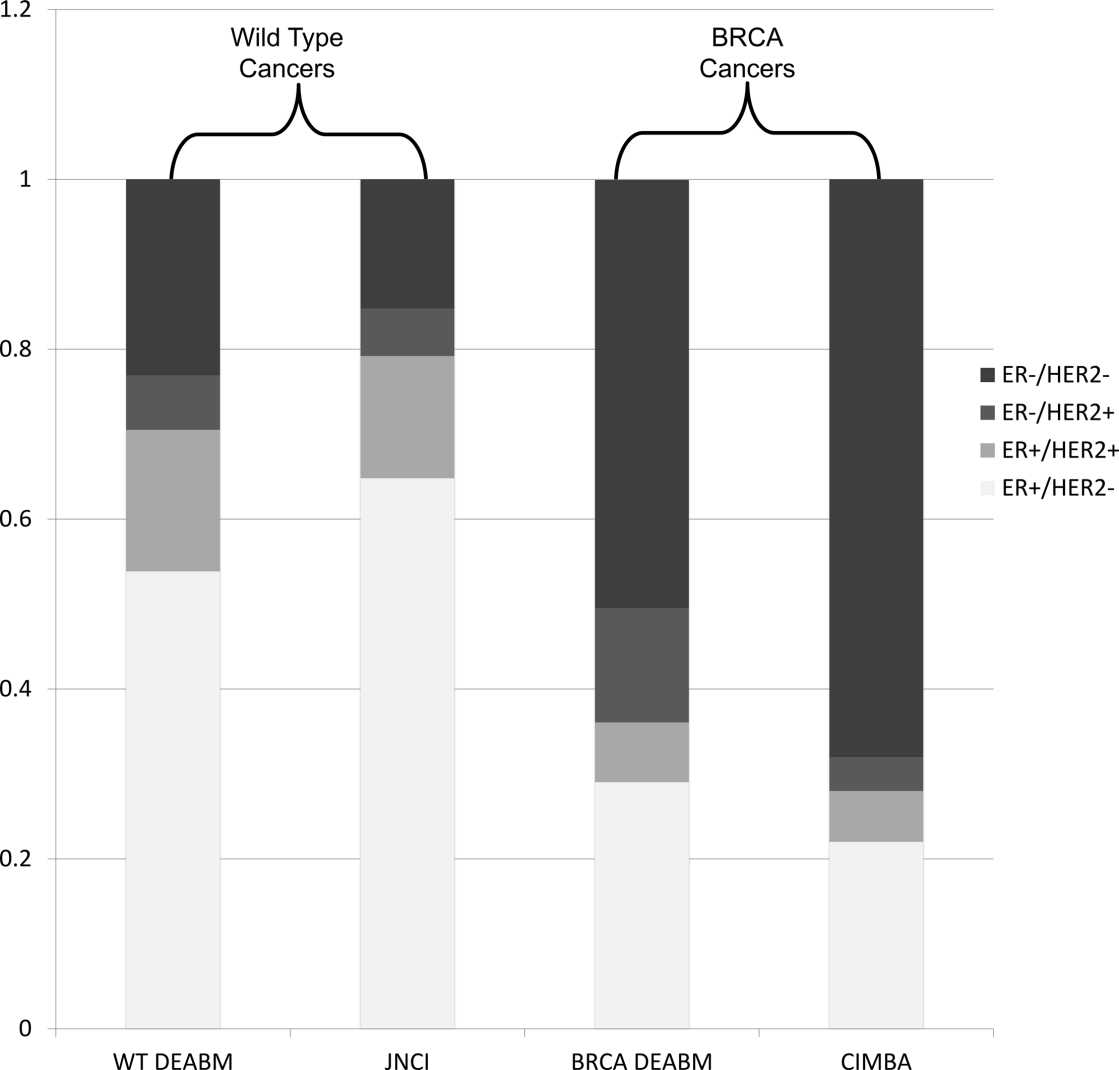
The proportion of tumors expressing ER and HER-2 in wild-type (WT) and BRCA1-mutated tumors generated by the Duct Epithelium Agent-based Model (DEABM). Individual tumors were grouped into one of four categories by combined estrogen receptor (ER) and HER-2 expression status. WT data from the DEABM is compared against data on ER and HER-2 status of breast tumors from the SEER database[45]. BRCA1-mutated data from the DEABM is compared against CIMBA data on ER and HER-2 status of tumors in a population of BRCA mutation carriers.[51]

**Fig 6:**
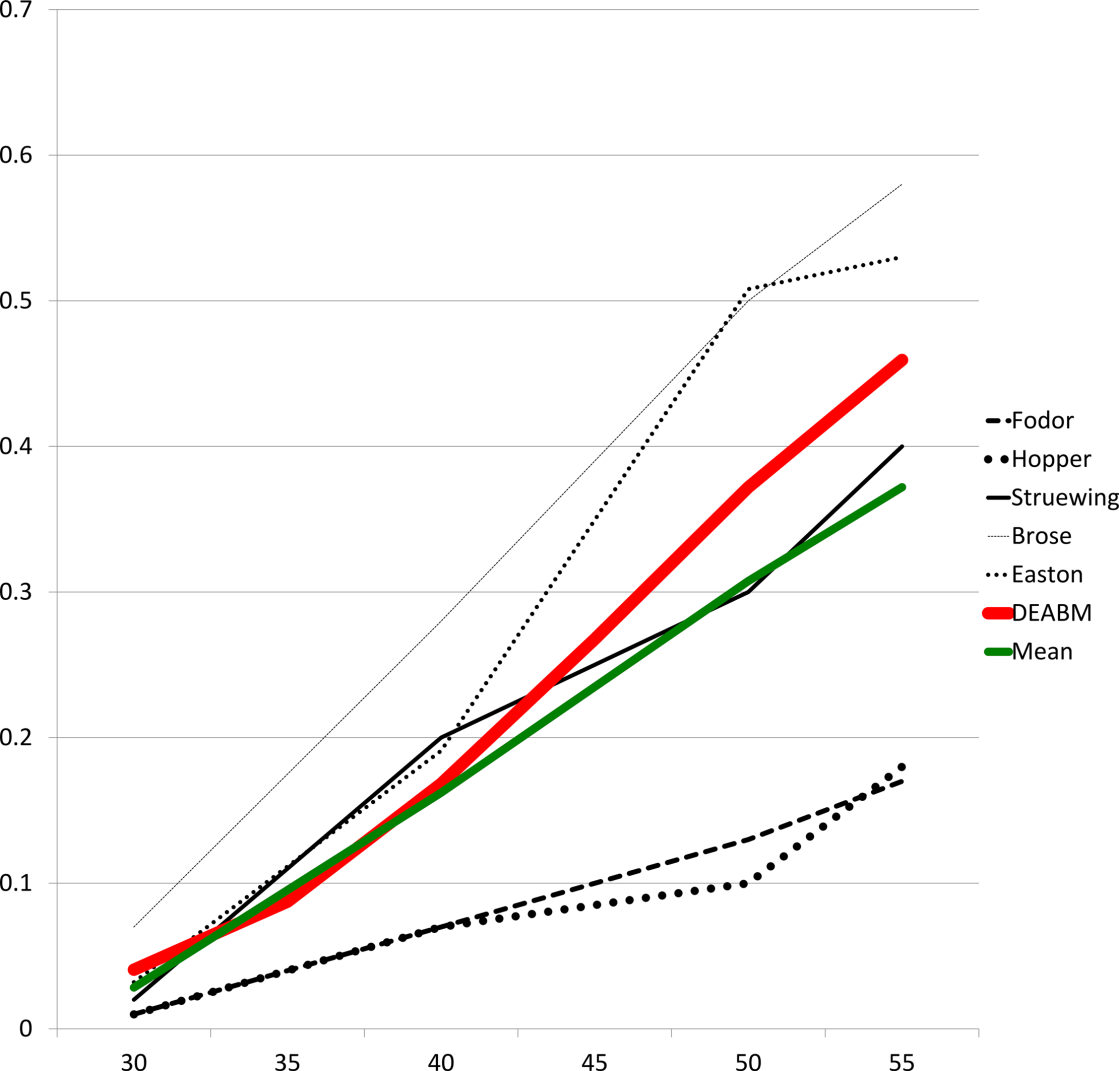
Cumulative cancer incidence over time of BRCA-1 mutated simulations in the Duct Epithelium Agent-based Model (DEABM) is compared to the results of five published studies of epidemiologic data. The cumulative incidence generated by the DEABM over 3,000 simulations was 45.9% by age 54 (95% CI 44.1–47.7%), a figure that fell within the range of published data (18%-58%) and compared favorably to the mean of the five studies (37.2%).

The DEABM generated tumors in both the wild-type and BRCA1-mutated conditions that closely matched known epidemiological rates. Incidence of tumor subtypes by ER and HER-2 status for wild-type and BRCA1 simulations are detailed in Fig 5. Tumor subtypes in each group were compared to observed rates in previously published epidemiological studies. Wild-type simulations had a cumulative cancer incidence of 2.6% by age 54 (95% confidence interval 2.0–3.2%), compared to the 2.9% in women by age 55 recorded in the SEER database [45]. BRCA1-mutated simulations had a cumulative incidence of 45.9% (95% confidence interval 44.1–47.7%), a value that falls within the range of five previously published studies on breast cancer incidence in BRCA-1 mutation carriers (17–53%) [46–50]. Cancer incidence over time in the DEABM is compared to epidemiologic data of breast cancer in BRCA-1 mutated patients in Fig 6.

In wild-type simulations tumors were 54% ER+/HER-2-, 17% ER+/HER-2+, 6% ER-/HER-2+ and 22% ER-/HER-2-. Tumors in the BRCA-1 mutated group were 29% ER+/HER-2-, 7% ER+/HER-2+, 13% ER-/HER-2+ and 50% ER-/HER-2-. Tumor subtype profile by ER and HER-2 expression were very similar to previously published data for both the wild-type and BRCA-1 mutated groups [45, 51]. These values are compared to epidemiological data in Fig 5.

### Analysis of early mutations by ER status of tumors:

**Fig 7:**
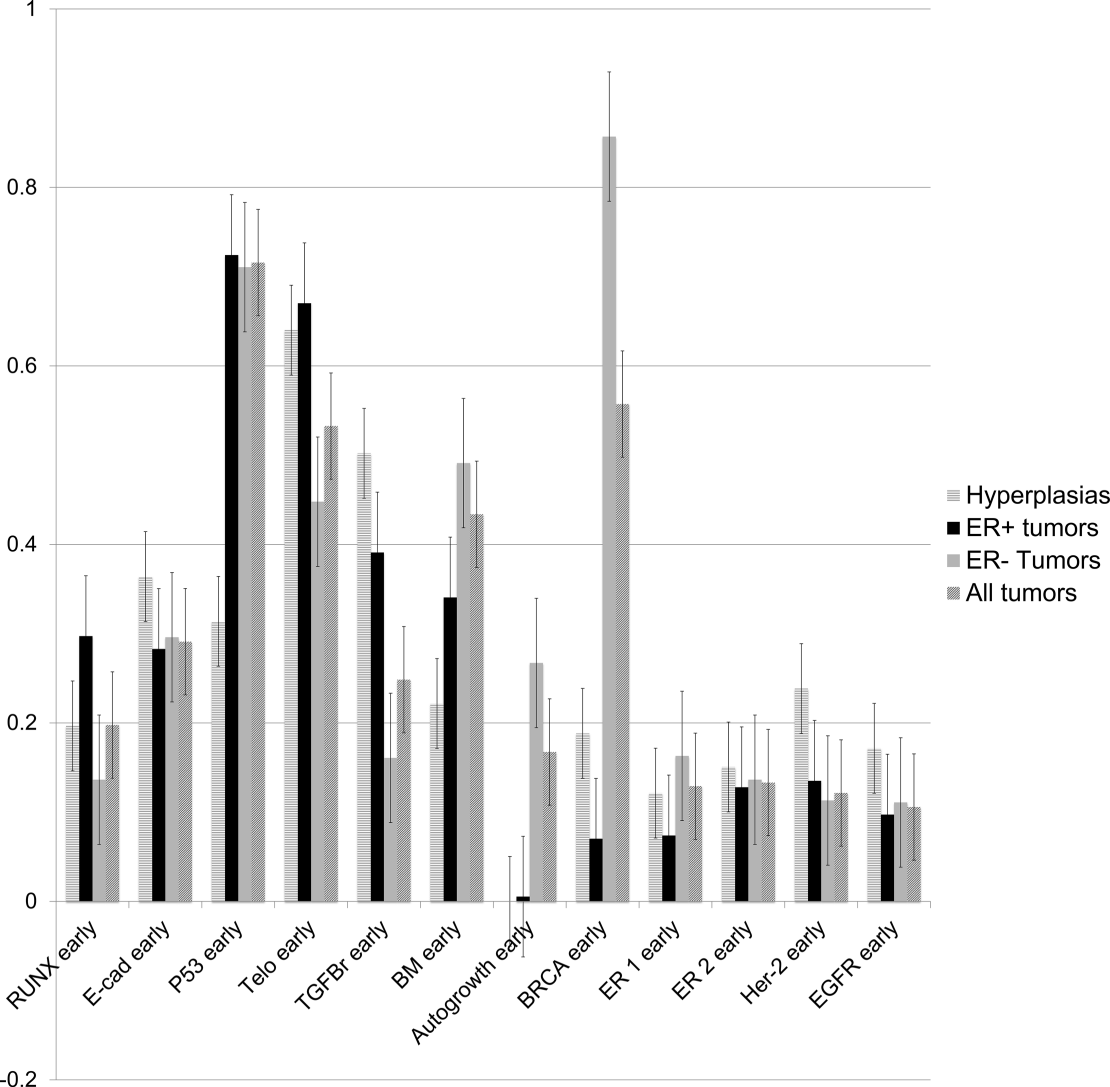
The proportion of hyperplasias, ER+ tumors, ER- tumors and a composite of all tumors bearing an “early” mutation in each of the Duct Epithelium Agent-based Model’s (DEABM) genes is represented above. The DEABM allows for the sequential tracking of mutations that have been acquired by a lineage of cells and a mutation was defined as “early” if it occurred as one of the first five mutations acquired. By unpaired, two-tailed T-test ER+ tumors accumulated more early mutations in genes leading to impaired apoptosis (telomerase, p < .01) and proliferation in response to estrogen (RUNX3, TGFB-r, p < .01). ER- tumors accumulated more early mutations in genes associated with genomic instability (BRCA1, p < .01), epithelial-mesenchymal transition (MMP-3, p < .01), and hormone-independent growth (c-MYC, p < .01). Early P53 mutations were common to all tumors. Hyperplastic populations were significantly more likely to acquire early mutations to telomerase, TGFB-r and E-cadherin, genes predisposing to failure of apoptosis and proliferation of ER+ cells without invasive behavior.

**Fig 8:**
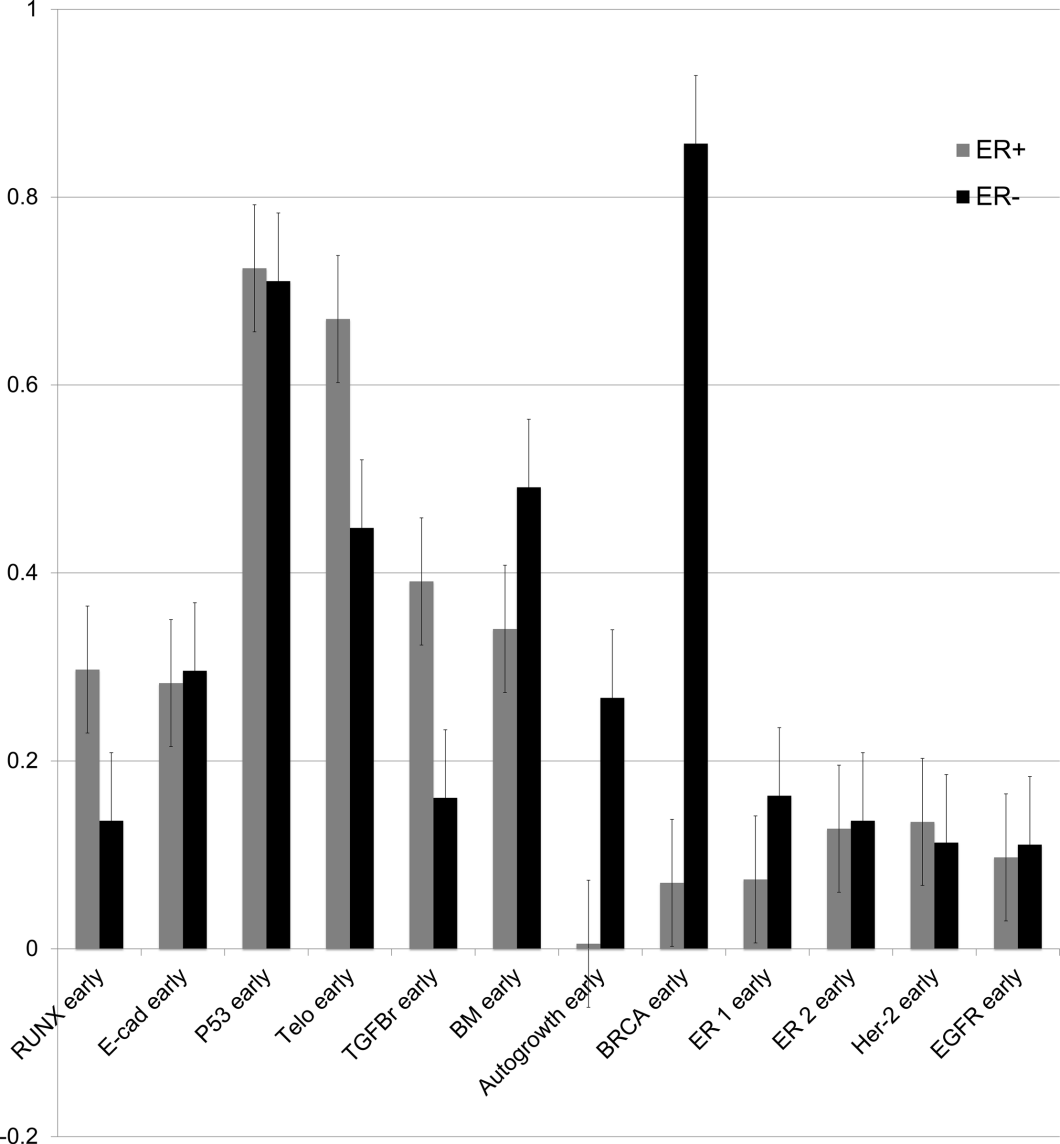
The proportions of cell lineages bearing “early” mutations in individual genes in ER+ and ER- tumors generated by the model are directly compared. Early mutations were defined as mutations that were among the first 5 acquired by a given cell lineage. By unpaired, two-tailed T-test ER- tumors accumulated more early mutations in genes associated with genomic instability (BRCA1, p < .01), epithelial-mesenchymal transition (MMP-3, p < .01), and hormone-independent growth (c-MYC, p < .01), corresponding to known data on increased genomic instability in ER- tumors and increased frequency of c-MYC expression. ER+ tumors accumulated more early mutations in genes leading to impaired apoptosis (telomerase, p < .01) and proliferation in response to estrogen (RUNX3, TGFB-r, p < .01), a pattern of early mutations that overlaps with that observed to be most likely in ER+ tumors.

The prevalence of early mutations between tumors was tested by two-tailed, unpaired T-test. ER+ tumors differed markedly from ER- tumors in the pattern of early mutation events. ER+ tumors were significantly more likely to accumulate early mutations in genes associated with impaired apoptosis (telomerase, p < .01) and proliferation in response to estrogen (RUNX3, TGFB-r, p < .01). ER- tumors were significantly more likely to accumulate early mutations in genes associated with genomic instability (BRCA1, p < .01), epithelial-mesenchymal transition (MMP-3, p < .01), and hormone-independent growth (c-MYC, p < .01). Early P53 mutations were common in all tumors [29, 52]. ER+ and ER- tumors did not vary significantly in the proportion having particular ER variant mutations, and both ER+ and ER- tumors carried ER variant mutations at a frequency near 10%. Frequencies of mutations to functional genes in the DEABM are detailed in Fig 7, comparison of mutation frequencies in ER+ vs ER- tumors is detailed in Fig 8.

### Frequency of genetic mutations: Malignancies versus hyperplastic lesions in the DEABM

**Fig 9:**
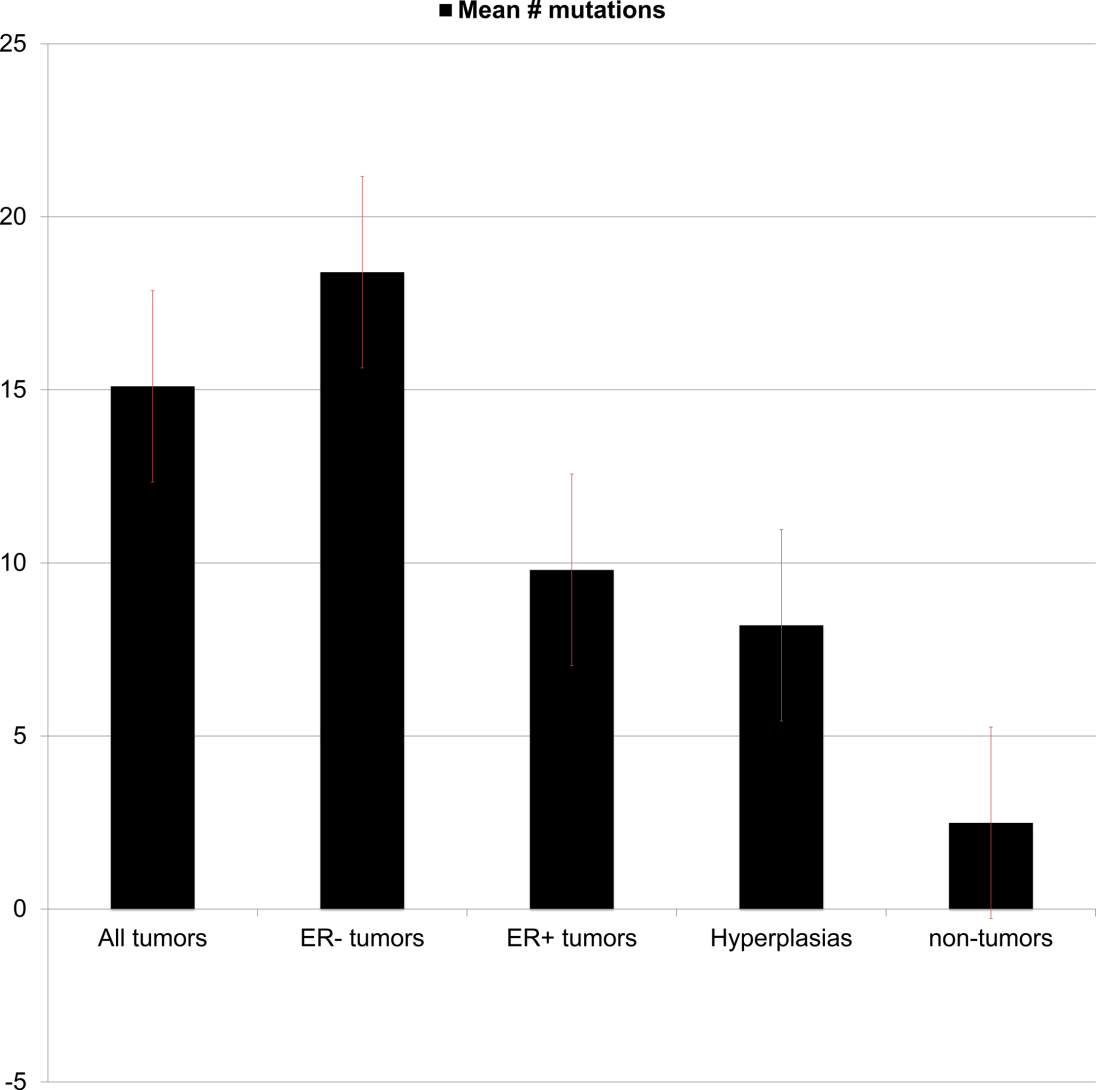
The total number of mutations acquired by tumors (ER+ and ER-), hyperplasias and normal populations generated by the model is depicted above. A mutation was defined as any event in which a dividing cell with DNA damage caused a copy number of a gene to be lost as detailed in the Methods section. By unpaired, two-tailed T-test ER- tumors acquired significantly more mutations than ER+ tumors (p < .01). Hyperplastic populations acquired significantly more mutations than normal populations (p < .01).

Mutations present in hyperplastic populations, defined as an expansion of the cellular population to greater than 2x normal, were similar to those present in ER+ tumors but not ER- tumors. 5.3% of simulations were “hyperplastic” at the end of the simulated period. The total number of mutations to functional genes accumulated by hyperplastic populations was significantly greater than in non-hyperplastic populations (8.2 vs 2.5, p>.01, Fig 9). Hyperplastic populations were more likely to carry mutations in telomerase, E-cadherin and TGFB-r, genes directly related to increased proliferative capacity and failure of apoptosis. As noted above, these mutations were also significantly more likely to be early mutations present in ER+ tumors, and have been observed to be common mutations in pre-malignant breast lesions (TGF-beta [53], telomerase p < .01 [54]).

ER- tumors were more likely to carry early mutations in BRCA1, MYC and genes associated with epithelial-mesenchymal transition (MMP-3, p < .01). ER- tumors acquired significantly more mutations than ER+ tumors (p < .01). This data matches observed data on increased genomic instability in ER- and BRCA1-associated tumors[55].

## Discussion

### A dynamic representation of current breast cancer knowledge

Here we demonstrate that the DEABM, a previously published and validated model of the mammary ductal epithelium, is able to generate diverse and heterogeneous breast cancer subtypes at rates that are similar to previously published epidemiological data. ER and HER-2 expression are among the most important clinicopathologic tumor characteristics, and the rates of receptor expression status in the DEABM closely match those observed in both wild type and BRCA-1 mutated populations. As would be expected, the majority of hyperplastic lesions carry mutational profiles resembling ER+ tumors. Conversely, ER- tumors developed early mutations that predisposed to genomic instability and a rapid rate of mutation and therefore transformation, which could be an added explanation for the paucity of clinically identifiable precursor lesions and interval cancers in ER—breast cancers and BRCA-1 mutation carriers.

Analysis of the functional genome of malignancies generated by the DEABM shows that ER+ and ER- tumors carry distinct patterns of genetic alterations, a phenomenon consistently observed in ER+ and ER- tumors *in vivo* [2]. ER+ tumors in the DEABM were significantly more likely to carry mutations that had functional consequences related to hormone-dependent growth and failure of cell death, whereas ER- tumors were more likely to carry mutations related to genomic instability and early invasive behavior. ER- tumors also accumulated a significantly greater number of mutations than ER+ tumors, a finding consistent with previously observed differences in the mutation rate between ER+ and ER- tumors [55]. An advantage of the DEABM is that it allows for the sequential tracking of the mutations acquired by malignancies, something not currently possible in cross-sectional genetic analyses of tumors. The data generated by the DEABM demonstrates that not only do the mutations present in ER+ and ER- tumors differ, but that the early, driving mutations are markedly different between these tumor subtypes. One explanation is that ER- tumors must engineer a way of proliferating without any hormonal stimulus, selecting for early mutations that confer hyperactivation of proliferation-promoting transcription factors that would allow a cell population to bypass the need for external hormonal signaling. For example, ER- tumors in the DEABM are more likely to carry mutations in the c-MYC oncogene, which confers increased risk of hormone-independent proliferation. This corresponds to data on c-MYC’s association with both ER- tumors, tumors with higher Ki-67 indexes and poor prognosis [56]. c-MYC lesions in the DEABM are also to occur early in the mutational history of ER- tumors, corresponding to data on c-MYC’s association with higher Ki-67 indexes, larger tumors and comedo-type in DCIS lesions, indicating that it may play an early role in fast-growing, high-risk DCIS lesions that are predisposed to becoming invasive [57, 58]. By focusing on key, early, driving mutations, an exciting possibility is that more advanced versions of the DEABM could help identify novel chemopreventive targets in the specific pathways leading to ER+ and ER—invasive breast cancers.

### The DEABM as a means to study tumor evolution

It has been frequently proposed that clonal expansion of cell lineages and cell selection may explain the genetic and phenotypic heterogeneity of breast cancer subtypes, a theory that is difficult to demonstrate because of the temporal and probabilistic difficulties posed by breast cancer pathogenesis [59]. The results generated by the DEABM provide further evidence for the plausibility of clonal evolution as a fundamental driving mechanism in breast cancer pathogenesis and subtype heterogeneity.

In this expansion of the DEABM we also demonstrate that it is able to generate plausible pre-malignant, hyperplastic states. Hyperplastic states in the DEABM behave in a mechanistically similar way to hyperplastic breast lesions, with expansions of cellular populations but without invasion beyond the basement membrane. The three variants of ER function attainable via functional mutations in the DEABM, as well as failure to suppress c-Met in ER+ cells, allow for the generation of non-invasive proliferative overgrowths of ER+ cells that are representative of many benign hyperplastic states. The inclusion of behavioral variants—representing a suite of alterations known to occur in ER function caused by epigenetic, splice site and genomic alteration[60]—allows for a more complex representation of ER+ tumors. It also makes possible a more thorough examination of the mechanisms behind how hyperproliferative, non-invasive populations may arise and serve as both a precursor state and a kind of “fertile ground” for the further mutations required for malignant transformation. Hyperplastic states generated by the DEABM, which are hyperproliferative secondary to genetic lesions governing key pathways that are also mutated in malignancy, clearly represent intermediate points on a continuum from normal tissue dynamics to the altered and unregulated dynamics that define malignancy.

The DEABM, and agent-based models in general, provide tools for analyzing the pathogenic mechanisms behind cancer that are impossible via traditional research methods. To put the model’s capabilities in perspective, it is effectively taking a tissue biopsy of the breast and performing genetic analysis on every cell, every day, for 40 years and in 3000 distinct individuals. To accomplish the analysis that the DEABM is able to perform, this would translate into 15,000 sequential biopsies per individual, or 45 million total samples for the entire 3,000 “patient” cohort, in order to sequentially track each of the mutations that occur over the entire experiment. Clearly, this is not feasible in traditional pre-clinical or clinical studies. Furthermore, the DEABM is able to identify the moment a pre-malignant state occurs and the moment the hyperplastic lesion transforms to cancer.

The DEABM is the first model of cancer that integrates the probabilistic nature of genetic insults and replication errors, gene-function interactions, intracellular molecular mechanisms, cell-cell interactions, and paracrine signaling between multiple cell types into a model of cancer pathogenesis and tumor evolution. Other published models of breast cancer have examined stem-cell hierarchies in DCIS [61], the pathogenesis of specific subtypes of DCIS[62], and the mechanical forces involved in DCIS tissue architecture [19], issues not explicitly treated by the DEABM. The DEABM’s ability to model the sequential, longitudinal changes that a cell population undergoes in the transformation from the dynamic state of health to malignancy is an important step in addressing the serious clinical problem of stratifying which premalignant lesions are at high risk of progressing to invasive cancer. Autopsy studies of the incidence of occult DCIS vary greatly; the largest series of Danish women aged 40–70 years found an incidence of occult DCIS of 39% [63]. Given the relatively low incidence of invasive breast cancer compared to DCIS, it is clear that not every case of DCIS will transform into invasive ductal carcinoma. Our current inability to predict which DCIS lesions are likely to progress results in significant overtreatment of DCIS [64, 65]. Traditional research methods have not yielded the answers to these difficult questions because they are not well suited to either the long time frame over which cancer develops or the rare and largely random genetic events that drive cancer progression. Because of the unique properties of agent-based models, the DEABM is an ideal tool for examining the process by which populations of normal ductal epithelial cells can transform into a diverse array of malignant phenotypes and genotypes via a process of clonal evolution driven by probabilistic mutational events. Interestingly, the role of stochastic processes (i.e. “bad luck”) in the generation of cancer has recently been further substantiated via mathematical modeling, albeit in an abstract, non-mechanistic fashion [66]. The vocal concerns raised by the cancer community regarding the interpretation of “bad luck”[67] could have been alleviated by a recognition of our previous incorporation of stochastic processes in the earlier version of the DEABM [18] and our more general model of the evolutionary dynamics of oncogenesis due to inflammation [35].

## Limitations

Because it is impossible to model every aspect of cancer biology, the DEABM necessarily treats certain aspects abstractly or incompletely. A limitation of the DEABM is that the post-menopausal state is not part of the model. The current state of knowledge regarding the physiology of the human breast in the perimenopausal, menopausal, and post-menopausal period is too limited to permit model development at this time. However, because of the long time course of cancer development and sequential accumulation of mutations [26], it is plausible that many post-menopausal breast cancers have their origin in the pre-menopausal period. Also, the DEABM does not generate tumors that recreate known cellular architecture of tumors or pre-malignant lesions, and therefore does not allow pre-malignant lesions to be separated into the traditional clinicopathologic categories (usual hyperplasia, atypical hyperplasia, and DCIS). However, this can be viewed as a strength of the model and highlights the notion that transformation from benign to malignant can occur at any time based on the type and number of mutations that occur in hyperplastic lesions. Another limitation is the current iteration of the model does not incorporate detailed pathways associated with PR and HER-2, and because ER regulates PR in our model, it cannot differentiate between ER+/PR+ and ER+/PR-tumors to more closely model what we clinically categorize as Luminal B tumors As the biology associated with estrogen is both clinically important, as well as the most extensively studied, we chose to focus on the biology of ER in this version of the DEABM. Lastly, the important effects of adipocytes and the immune system on breast cancer biology are not modeled by the DEABM [11].

It is infeasible to comprehensively model the approximately 20,000 human genes, their multiple protein products that arise from transcription, RNA splicing and translation, and the complex interactions of every protein both within and between cells. Further, important and interesting risk factors for the development of breast cancer such as parity, breastfeeding, obesity, and others—for which the biology remains largely unknown—were beyond the present scope of the present investigation. The goal of the DEABM is rather to facilitate a more global examination of the fundamental driving mechanisms behind cancer—the emergent behaviors of aberrant cellular populations that arise out of altered functional pathways, pathway alterations that are themselves the product of probabilistic insults to DNA. The explicit goal of the DEABM is to examine mechanisms behind the transition from healthy breast tissue to invasive cancer. The abstractions employed in the DEABM enhance this focus on the larger picture, and demonstrate that abstract treatments of complex processes can be used to accurately represent global biology, and how transitions from states of health to disease might occur. The DEABM’s simplified genome, focusing on a relatively small number mutations of with large effects on cellular behavior is consistent with observations that while cancers contain large amounts of mutation, as few as three “driver mutations” may be required for malignant transformation [68]. The ability to model complex phenomena via abstracted models reinforces the implications of the nested nature of biological networks and the overall robustness of biological systems. Because the DEABM is the first attempt to model the multi-scale nature of breast cancer pathogenesis, it was designed to be a minimally sufficient model to accomplish this goal. This approach is consistent with the modeling and simulation paradigm of progressive validation; as iterative model development progresses, additional detail can be included [69, 70].

### Agent-based modeling and the future of breast cancer research

Despite the inherent limitations of computational models of biologic systems, we believe that the DEABM, and ABMs more generally, represent invaluable tools in the biology of complex disease processes. ABMs allow for the *in silico* examination of phenomena that are infeasible via more traditional research methods. This is relevant to the increasingly emerging view that while environmental and genetic risk factors are important in determining cancer risk, much of the process that leads to cancer is inherently probabilistic [66]. As an analytic tool, the DEABM can track the exact sequence of mutations acquired by a tumor along the trajectory of its natural history from normal to malignancy. By analogy, the DEABM allows for *in silico* cohort studies of cellular populations, as compared to the cross-sectional nature of tissue studies that analyze a tumor’s genetic profile at a specific point in time.

The current state of ambiguity concerning the risk potential of premalignant breast lesions, as well as how premalignant lesions should be clinically managed, is undesirable[71]. A recent study by Elmore and colleagues demonstrated that the agreement rate among breast pathologists in cases of cellular atypia was just 48%[72], which is troubling given that a diagnosis of atypical ductal hyperplasia carries with it a recommendation for follow-up surgical excision, since some 15–30% of these lesions harbor an occult cancer [73, 74]. In response to this situation—on in which important decisions are made with inconsistent information—a follow-up editorial by Davidson and Grimm called for increased research into molecular markers to augment diagnosis and clarify risk in atypia and other pre-malignant breast lesions [75]. By further understanding the biology of pre-malignant lesions—how they functionally and genetically relate to both normal tissue and invasive cancer—clinicians might be better able to counsel patients regarding their risk of developing invasive cancer and avoid treating patients who will not progress. The DEABM’s examination of pre-malignant states is consistent with a continuum model of tumorigenesis—that cancers arise out of an accumulation of genetic abnormalities over time, and that these cumulative genetic changes would be evident and observable by more detailed assessment of the tissue at risk. In the the hyperplastic lesions produced by the DEABM, we find similar genetic lesions to those that are “early” lesions in ER+ cancers generated by the model. Based on the known oncogenic mechanisms encoded in the DEABM we would hypothesize that tissue samples of clinically relevant pre-malignant lesions might show a similar pattern of genetic markers. This concept, which extends the current thinking concerning “tissue-at-risk” regarding oncogenic potential, points toward a future where premalignant lesions will be characterized by their mutational profile and we will move beyond histologically based treatment decisions.

Agent-based models like the DEABM also address the important issue of tumor heterogeneity. While pathologic parameters such as ER and HER2 status are important prognostic indicators, there is still substantial variability in the response rate to targeted therapies [76–80]. This variability arises out of the multiple different trajectories that tumors can take to similar pathologic endpoints. The DEABM allows for tumors with identical ER or HER2 status that arise via different pathways and sequential mutations. Understanding the mechanisms that drive a tumor will be essential in understanding why tumors with identical prognostic indicators respond to targeted therapies in different ways, or develop resistance at different rates [81]. The DEABM allows for the sequential tracking of mutations present in a given cell, and thus can be used to map the progressive transformation of the cell’s genetic and functional change.

While the DEABM and other agent-based models do not themselves create empirical data, they can generate and provide a first level of validation for new hypotheses that push research forward in new directions [82]. Traditional research methods have generated enormous amounts of genetic and mechanistic data about breast cancer biology; the clinical utility of much of this information is yet to be discovered. One of the chief challenges in making such data useful is in contextualizing such information into mechanistically plausible, interacting sequences of events that lead to recognizable, plausible biologic behaviors of cellular systems [15]. The DEABM, by integrating diverse basic science mechanisms into a functional model of the mammary epithelium’s latent ability to transform from a stable, “healthy” system into an unregulated, malignant behavior pattern, at rates and with tumor markers that mimic the epidemiology of breast cancer, is an example of how steps might be taken in this direction. Dynamic representation of breast cancer biology via computational models like the DEABM makes it possible to generate functional maps of the current state of knowledge, and to use these maps to explore complex aspects of biologic basis of disease. We believe that in studying complex, highly interactive and fundamentally probabilistic diseases like breast cancer, new methods like the DEABM can be helpful tools in both the process of hypothesis generation and as adjuncts to traditional research methods.

## Supporting Information

S1 Text Supplemental Methods. (DOCX)

## Abbreviations

ABM: Agent-based model
DEABM: Duct Epithelium Agent-based Model
DCIS: *Ductal carcinoma in situ*
ER: Estrogen receptor
PR: Progesterone receptor
