# Supplementary material for: Examining the Relationship between Pre-Malignant Breast Lesions, Carcinogenesis and Tumor Evolution in the Mammary Epithelium Using an Agent-Based Model
Source: PLoS One. 2016 Mar 29;11(3):e0152298. doi: 10.1371/journal.pone.0152298 (PMC4811527; doi:10.1371/journal.pone.0152298)
Supplement: S1 Text — (DOCX) [file pone.0152298.s001.docx]

**DEABM Supplemental Methods:**

The development, calibration and mechanistic details are described extensively in Chapa et al 2013, the article that first presented and validated the DEABM as a mechanistic representation of mammary epithelium dynamics[1]. Provided below are more comprehensive descriptions of the DEABM’s methods in than provided in the present article’s main text and are intended to augment the present article. Any new mechanisms included in the present version of the DEABM are described in full below, with applicable references. Mechanisms left unchanged from the DEABM in our previously published paper are noted below, full details and applicable supporting citations can be referenced in the Methods of Chapa et al 2013. The complete code of the DEABM can be downloaded at the following link: <http://bionetgen.org/SCAI-wiki/index.php/Main_Page>

**--Environment**

*Spatial Representation in the DEABM:*

The DEABM represents a two-dimensional patch of bilayered mammary ductal epithelium. Conceptually, the DEABM can be pictured as a portion of duct split open and laid flat. Space is represented in a two-dimensional, toroidal square grid populated by motile agents representing cells, and immotile (fibroblasts) agents.  Grid spaces (“patches” in NetLogo terminology) in the DEABM allow for both space that cells occupy, as well as spaces to abstractly represent extracellular concentrations of hormones, mediators and the basement membrane.

The DEABM does not attempt to accurately recreate all aspects of spatial effects in the 3-dimensional environment of the breast. The space represented by the DEABM is better viewed as a representation of the cellular interaction/communication network rather than as an attempt to replicate histological detail. The spatial representation and scale of the DEABM, as well as discussion of the DEABM with respect to other ABMs that seek to represent spatial aspects of the breast can be found in the methods of Chapa et al 2013.

*Time:*

During simulations the DEABM progresses forward in single step increments, with each step representing one day of simulated time. At each step, state and environmental variables are updated, local growth factors and hormones diffuse, DNA damage is accumulated and repaired, and agents execute the algorithms governing their behavior. The one-day timescale was chosen to reflect the length of the cell-cycle period observed in mammary epithelial cell division [2-4]. Estrogen and progesterone are updated with each time-step, with the levels set based on published values for a 28-day cycle at baseline. [5-8].

**--Cell Types**

The DEABM comprises fixed cells representing fibroblasts as well as motile cells representing ductal luminal cells, myoepithelial cells and their respective progenitor and stem cells.

Fibroblasts: Fibroblasts in the DEABM are represented by non-motile agents and their function is heavily abstracted. Functional aspects of fibroblasts in the DEABM are focused on their role supporting the epithelial life cycle and organization. Fibroblasts are involved in the paracrine signaling described in figure 1 in the main text. The function of fibroblasts in the DEABM is unchanged from that described in Chapa et al 2013.

*Myoepithelial Cells:*

Myoepithelial cells are basal cells that adhere to the basement membrane, provide structural support to the breast duct and are involved in milk secretion. In the DEABM their primary role is to secrete and maintain the basement membrane, which functions as a regulator of the local growth and spread of luminal cells.

Myoepithelial cells do not accumulate or repair DNA damage, and thus do not mutate or have malignant potential in the DEABM. The function of myoepithelial cells and the algorithms governing their functional behaviors are unchanged from that described in Chapa et al 2013. Proliferation of myoepithelial cells is governed by the intracellular signaling mechanisms described above.

*Myoepithelial progenitor cells:*

Myoepithelial progenitor cells can arise from division of a myoepithelial stem cell or a myoepithelial progenitor cells, as well as via mesenchyal transition of a differentiated myoepithelial cell in response to high levels of RANK ligand [9].

Myoepithelial progenitor cells have five days to find an appropriate patch and differentiate; cells not finding adequate position undergo apoptosis.

*Luminal Cells:*

Luminal cells are represented as motile cells overlying both fibroblasts, myoepithelial cells and the basement membrane. Luminal cell subtypes include luminal stem cells as well as luminal progenitor cells, which can arise as the product of either asymmetric stem cell division or luminal cell mitosis.

Luminal cells are capable of expressing both the estrogen receptor and progesterone receptor. Under normal conditions ER expression is determined upon the birth of a daughter cell, with cells having a 10% probability of expressing ER, a number corresponding to physiologic levels in breast tissue [10-15]. A probability function was chosen to dictate ER expression because the mechanisms determining ER expression in normal luminal cells remains unknown. PR expression is controlled by ER expression, as it is in the normal mammary epithelium [16]. Mechanisms by which this “normal” pattern of determining ER expression can be altered by genetic mutations are outlined in the section outlining the functional genome of the DEABM and in Chapa et al 2013.

*Luminal Progenitor cells:*

Luminal progenitor cells are the undifferentiated products of luminal cell mitosis. They are motile, unadhered to the basement membrane and are capable of expressing ER and PR via the same mechanisms described above for differentiated luminal cells. Luminal progenitor cells are also capable of undergoing mitosis, and adhere to the same rules for triggering the cell cycle, as well as cell cycle inhibition as described for luminal cells above.

Luminal progenitors have 5 days to find a patch of space that has not reached carrying capacity with a present basement membrane. If these conditions are met the progenitor cell will differentiate into a luminal cell and adhere. If not, the cell will undergo apoptosis. For a full explanation of the methods governing luminal progenitor behavior and the underlying supporting mechanisms, please see the “Methods” section of our earlier paper “Examining the Pathogenesis of Breast Cancer Using a Novel Agent-Based Model of Mammary Ductal Epithelium Dynamics”[1]

*Stem Cells:*

Luminal and myoepithelial lineages in the DEABM arise from distinct stem cell populations[17, 18]. The DEABM does not seek to model the details of mammary stem cell regulation and life cycle; stem cells in the model function as a renewing source of progenitor cells. Luminal stem cells in the DEABM can acquire mutation and undergo DNA damage and repair. Acquired mutations are subsequently inherited by daughter cells. Simulations begin with 4 stem cells of both the luminal and myoepithelial lineages. Stem cell divisions are asymmetric and probabilistic, with every day yielding a 25% chance of stem cell division. Stem cell division leads to a single daughter progenitor cell and persistence of the parent stem cell. The functions of stem cells are unchanged from Chapa et al 2013.

**Paracrine signaling:**

Cell-cell signaling in the DEABM is mediated by abstracted levels of local growth factors that are produced and absorbed by cellular agents. Environmental (“Patch”) variables are used to conceptually represent concentrations of local growth factors available to cells occupying a patch of space in the DEABM. The hormones and growth factors represented in the DEABM include: amphiregulin, hepatocyte growth factor (HGF), RANK-ligand and transforming growth factor beta (TGF-β). These variables and the parameter values associated with them are detailed in supplementary table 1. Cells produce and absorb these factors via the mechanisms detailed in Chapa et al 2013.

**Intracellular signaling**

Proliferation in the DEABM is governed by a mechanism combining systemic hormonal, local paracrine and intracellular signaling mechanisms (see figure 2 in main text). In the normal state of the DEABM, estrogen stimulates the secretion of amphiregulin, which in turns stimulates fibroblasts to secrete HGF. Cells possessing the c-met receptor for HGF are able to respond to present growth factor. HGF causes a rise in the internal production of AKT, meant serves as an abstract representation of the pro-prolifeative activation of the PI3K/AKT/mTor pathway, with the rise directly proportional to the amount of HGF received by the cell. Cells also possess receptors for TGF-beta, a growth inhibiting hormone in the breast [19-22]. Presence of TGF-beta on a patch occupied by a cell causes a decrease in intracellular AKT level proportional to the amount of TGF-beta received. Intracellular AKT levels are thus dynamically result from a combination of global estrogen and progesterone levels and local paracrine signaling of both growth promoting and inhibiting factors. Cells that finish a day with an AKT level greater than “AKT-threshold” will divide, given that other proliferation conditions are met (e.g. Hayflick number <40, absence of senescence state, sufficient space to allow cellular division).

Cells must possess receptors to respond to local growth hormones, and receptor expression is governed by the functional genome of the DEABM. By default, normal ER- cells in the DEABM are (HGF+/EGFR-), whereas normal ER+ cells in the DEABM are (HGF-/EGFR-). Mutations to genes regulating the expression of receptors can cause cells to express receptor combinations that differ from the default, resulting in different behaviors in response to local growth factors.

**DNA Damage/Repair/apoptosis**

Mechanisms of DNA damage and repair are modeled abstractly and probabilistically in the DEABM. All luminal cells possess a variable “DNA-integrity”, which is initially set to an arbitrary value of 1,000 at the beginning of simulations and represents the normal state. Each day of simulated time in the DEABM, luminal cells accumulate DNA damage according to a probabilistic funtion. Similarly, algorithms specify a probabilistic mechanism by which DNA damage is repaired with each time step. A schematic representation of the DNA damage/repair and cell fates in response to DNA damage and mutations can be seen in Figure 3 in the main text. Cells’ DNA repair algorithms are modifiable via mutations to genes known to control these processes (BRCA1, P53). Loss-of-function mutations reduce cells’ ability to repair DNA damage and increase the probability of further mutations. The mechanisms outlining the concepts of DNA-integrity, senescence and apoptosis in response to high levels of DNA damage are outlined in detail in Chapa et al 2013.

**Apoptosis:**

All non-stem cells in the DEABM have an internal variable “bax-level” that functions as the mechanism controlling apoptosis. With the passage of each day cells set their “bax-level” higher by 1, and bax-levels continually rise with time. Differentiated cells undergo apoptosis upon the bax-level reaching 60, in accordiance with the observed life span of human ductal cells. Progenitor cells undergo apoptosis after 5 days if they do not differentiate, as outline below. Full details on apoptosis mechanisms, and mutations affecting apoptosis in the DEABM can be found in Chapa et al 2013.

**Mutations**

Cells undergoing mitosis with unrepaired DNA damage after leaving cell cycle arrest (i.e. with a “DNA-integrity < 100% but > 97.5%) acquire mutations represented by the agent-variable “new-mutations.” The value of “new-muations” is set to the difference between the cell’s “DNA-integrity” variable and 1000.  Each “new-mutation” is randomly assigned a number between 1-10,000, which represents the particular area of the genome that the mutation affects. Mutations that fall within regions of the DEABM’s genome containing one of the functional genes in the DEABM cause a copy number loss or gain of that gene. Mutations that fall in regions of the genome that do not code for functional genes are “silent” mutations, and have no effect. In the DEABM’s abstract genome, location sites 1-60 are occupied by functional genes, meaning that any given mutation has a 0.6% chance of affecting a functional gene, thus the vast majority of mutations are “silent”, as they are in biologic systems *in vivo.*

**Heritability in the DEABM:**

Daughter cells that are the product of mitosis inherit the genome and any acquired functional mutations of their parent cell.  This allows for clonal lineages of cells in the DEABM, as well as the divergence of lineages leading to heterogeneity of populations within a single simulation of the DEABM.

**The functional genome of the DEABM:**

1. Telomerase: The function of telomerase in the DEABM is to govern cellular senescence and proliferative capacity. Cells that acquire gain-of-function mutations of telomerase are able to divide beyond their Hayflick limit and achieve immortality. The function of telomerase is unchanged from that described in Chapa et al 2013.
2. E-cahedrin: E-cadherin governs cell-cell adhesion and apoptosis in the DEABM. Cells in the DEABM are required to be adhered to both the basement membrane and adjacent cells. Further cell-cell adhesion is required for progenitor cells to differentiate. Failure of E-cadherin delays the initiation of apoptosis in non-adhered cells. The function of E-cadherin in unchanged from that described in Chapa et al 2013.
3. TGF-β-receptor: Binding of TGF-β to the cell’s TGF-β-receptor function to inhibit proliferation via the intracellular signaling mechanisms described above. TGF-β-receptor activation is also necessary for the expression of RUNX3 (see below). Loss of TGF-β-receptor leads to reduced growth inhibition.
4. p53: p53 is involved in promoting the DNA repair, cell cycle arrest due to accumulated DNA damage and the initiation of apoptosis. P53 is also required for cells to differentiate into ER+ luminal cells [23-25]. Loss of p53 leads to impairment of all its associated functions.
5. Myc: Myc is a transcription factor and oncogene, overexpression of which increases the likelihood of cell division and promotes proliferation. At baseline in the DEABM, Myc is suppressed. Cells in the DEABM may acquire mutations leading to overexpression, which leads to increased intracellular levels of AKT and thus increased probability of proliferation.
6. Regulation of matrix metalloproteinases (MMP): MMPs are involved in cleaving extracellular matrix proteins and are critical in invasion beyond the basement membrane. Under baseline conditions in the DEABM, MMPs are regulated, preventing cells from invading beyond the basement membrane. Mutations that lead to loss of control of MMPs simulate epidermal-mesenchymal transition and remove the movement restriction on luminal cells, allowing them to grow and divide beyond the basement membrane.
7. BRCA1: BRCA1 function in the DEABM is required for DNA repair, entry into senescence and ER expression. Loss of BRCA function affects DNA damage repair, as well as a damaged cell’s entry into cell cycle arrest. It’s function is complementary to P53.
8. RUNX3: RUNX3 function in the DEABM is described in detail in Chapa et al 2013. It’s function in the DEABM is as a proposed mediator of c-Met expression and as an upstream regulator of ER expression. c-Met is required for proliferation and loss of c-Met suppression allows ER+ cells to proliferate in response to HGF. Failure of RUNX3 is a proposed mechanism by which ER+ cells can potentially proliferate and thus become hyperplastic populations and invasive cancers.
9. EGFR: EGFR is the receptor for epidermal growth factor, represented by amphiregulin in the DEABM. At baseline, luminal cells in the DEABM do not express EGFR and thus do not respond by increasing intracellular AKT levels in response to local amphiregulin. Cells can acquire mutations leading to a loss of suppression of EGFR, allowing for proliferation in response to amphiregulin.
10. HER-2: HER-2 is a ligandless receptor of the EGFR family and is known to be an important driver of proliferation in a minority of breast cancers[26]. In the default state of the DEABM luminal cells do not express HER-2. Mutations can suppress inhibition of HER-2, allowing for expression. HER-2 is believed to augment the proliferative response to EGFR ligands and in the DEABM it doubles the proliferative response to amphiregulin, thus promoting proliferation.
11. ESR1: Under baseline conditions the estrogen receptor in the DEABM has the function of responding to available estrogen and producing amphiregulin in proportion to received estrogen. Mutations affecting the estrogen receptor can have three different affects, corresponding to known genomic and non-genomic mechanisms involving the estrogen receptor implicated in breast cancer. These mutation effects could represent either genomic mutations to ESR1[27], epigenetic alterations in ESR1 expression and function[28], or mutations causing splice site alterations leading to altered ER function[29], all of which are mechanisms implicated in altered ER function in breast cancer:
12. Autonomous ESR1-mediated production of growth factor. In response to this mutation to this variant form of ESR1, cells produce a fixed amount of amphiregulin each day regardless of the level of endogenous estrogen.
13. Non-genomic promotion of proliferation: This variant form of ESR1 engages in cross-talk with growth factor receptors, in which estrogen-stimulated ER causes activation of the intracellular domain of growth factor receptors EGFR and c-Met, a mechanism known to be important in breast cancer and hormone-therapy resistance[30]. In the DEABM estrogen-stimulated ER directly increases intracellular AKT and thus promotes proliferation.
14. The third variant is a combined form of types I and II. This variant ER produces amphiregulin autonomously and increases intracellular levels of AKT.

**Parameter values:**

**Supporting Materials S1: Table of Parameter Values and Parameter Determination Process**

| **Parameter Type** | **Parameter** | **Value** | **Description** | **Fitted?** |
| --- | --- | --- | --- | --- |
| *Diffusion Rates of Secreted Mediators (Netlogo Function for Environmental Variables)* |  |  |  |  |
|  | Amphiregulin-level | 0.8 | Extracellular level of amphiregulin | No |
|  | HGF-level | 0.8 | Extracellular level of HGF | No |
|  | TGFB-level | 0.8 | Extracellular level of TGFB | No |
|  | RANK-level | 0.8 | Extracellular level of RANK | No |
| *Activity Thresholds (Agent Variable)* |  |  |  |  |
|  | Hayflick-number | 40 max | Telomer Length, limits number of cellular divisions | No |
|  | DNA-integrity | 1000 max | Counter for total amount of genetic damage, mutation rate is 1/1000 per time step | No |
|  | Bax-Level | 60 | Activates apoptosis | Yes |
|  | HGF-threshold | 50 | Amount of HGF required to trigger mitosis | Yes |
|  | TGFB-threshold | 250 | Amount of TGF-beta required to inhibit mitosis | Yes |
|  | Rank-threshold | 500 | Amount of Rank required to trigger mitosis | Yes |
|  | Senescence-Threshold | 975 | DNA-integrity levels below threshold trigger senescence in normal cells | Yes |
|  | AKT-threshold | 200 | AKT levels allow mitosis to proceed if other conditions have been met | Yes |
| *Binding/Consumption*  *(Agent-variable)* |  |  |  |  |
|  | Amph-uptake-max | 150 | Maximum amount of amphiregulin removable from the environment by a cell | No |
|  | HGF-uptake-max | 150 | Maximum amount of HGF removable from the environment by a cell | Yes |
|  | TGFB-uptake-max | 350 | Maximum amount of TGF-beta removable from the environment by a cell | Yes |
|  | Rank-uptake-max | 100 | Maximum amount of Rank removable from the environment by a cell | Yes |

This table lists key parameter values present in the DEABM. The column entitled “Fit?” denotes as to whether the parameter value was adjusted during the calibration process. Full description of the stages of calibration of the DEABM, fitting of variable parameters and general principles of model development specifically pertaining to the development of the DEABM are fully detailed in the supplemental table accompanying Chapa et al 2013.

1. Chapa J, Bourgo RJ, Greene GL, Kulkarni S, An G: **Examining the pathogenesis of breast cancer using a novel agent-based model of mammary ductal epithelium dynamics**. *PloS one* 2013, **8**(5):e64091.

2. Larsson O, Blegen H, Wejde J, Zetterberg A: **A cell cycle study of human mammary epithelial cells**. *Cell biology international* 1993, **17**(6):565-571.

3. Gautschi JR, Schindler R, Hurni C: **Studies on the division cycle of mammalian cells. V. Modifications of time parameters by different steady-state culture conditions**. *The Journal of cell biology* 1971, **51**(3):653-663.

4. Meyer JS, Connor RE: **Cell proliferation in fibrocystic disease and postmenopause breast ducts measured by thymidine labeling**. *Cancer* 1982, **50**(4):746-751.

5. Stricker R, Eberhart R, Chevailler MC, Quinn FA, Bischof P, Stricker R: **Establishment of detailed reference values for luteinizing hormone, follicle stimulating hormone, estradiol, and progesterone during different phases of the menstrual cycle on the Abbott ARCHITECT analyzer**. *Clinical chemistry and laboratory medicine : CCLM / FESCC* 2006, **44**(7):883-887.

6. Soldin OP, Guo T, Weiderpass E, Tractenberg RE, Hilakivi-Clarke L, Soldin SJ: **Steroid hormone levels in pregnancy and 1 year postpartum using isotope dilution tandem mass spectrometry**. *Fertility and sterility* 2005, **84**(3):701-710.

7. O'Leary P, Boyne P, Flett P, Beilby J, James I: **Longitudinal assessment of changes in reproductive hormones during normal pregnancy**. *Clinical chemistry* 1991, **37**(5):667-672.

8. Hankinson SE, Willett WC, Manson JE, Colditz GA, Hunter DJ, Spiegelman D, Barbieri RL, Speizer FE: **Plasma sex steroid hormone levels and risk of breast cancer in postmenopausal women**. *Journal of the National Cancer Institute* 1998, **90**(17):1292-1299.

9. Haslam SZ, Drolet A, Smith K, Tan M, Aupperlee M: **Progestin-regulated luminal cell and myoepithelial cell-specific responses in mammary organoid culture**. *Endocrinology* 2008, **149**(5):2098-2107.

10. Shoker BS, Jarvis C, Clarke RB, Anderson E, Hewlett J, Davies MP, Sibson DR, Sloane JP: **Estrogen receptor-positive proliferating cells in the normal and precancerous breast**. *The American journal of pathology* 1999, **155**(6):1811-1815.

11. Soderqvist G, von Schoultz B, Tani E, Skoog L: **Estrogen and progesterone receptor content in breast epithelial cells from healthy women during the menstrual cycle**. *American journal of obstetrics and gynecology* 1993, **168**(3 Pt 1):874-879.

12. Khan SA, Yee KA, Kaplan C, Siddiqui JF: **Estrogen receptor alpha expression in normal human breast epithelium is consistent over time**. *International journal of cancer Journal international du cancer* 2002, **102**(4):334-337.

13. Umekita Y, Souda M, Ohi Y, Rai Y, Sagara Y, Sagara Y, Yoshida H: **Expression of estrogen receptor alpha and progesterone receptor in normal human breast epithelium**. *In Vivo* 2007, **21**(3):535-539.

14. Taylor D, Pearce CL, Hovanessian-Larsen L, Downey S, Spicer DV, Bartow S, Pike MC, Wu AH, Hawes D: **Progesterone and estrogen receptors in pregnant and premenopausal non-pregnant normal human breast**. *Breast cancer research and treatment* 2009, **118**(1):161-168.

15. Sharma P, Kimler BF, Warner C, Metheny T, Xue Q, Zalles CM, Fabian CJ: **Estrogen receptor expression in benign breast ductal cells obtained from random periareolar fine needle aspiration correlates with menopausal status and cytomorphology index score**. *Breast cancer research and treatment* 2006, **100**(1):71-76.

16. Clarke RB, Howell A, Anderson E: **Estrogen sensitivity of normal human breast tissue in vivo and implanted into athymic nude mice: analysis of the relationship between estrogen-induced proliferation and progesterone receptor expression**. *Breast cancer research and treatment* 1997, **45**(2):121-133.

17. Visvader JE: **Keeping abreast of the mammary epithelial hierarchy and breast tumorigenesis**. *Genes & development* 2009, **23**(22):2563-2577.

18. Van Keymeulen A, Rocha AS, Ousset M, Beck B, Bouvencourt G, Rock J, Sharma N, Dekoninck S, Blanpain C: **Distinct stem cells contribute to mammary gland development and maintenance**. *Nature* 2011, **479**(7372):189-193.

19. Sternlicht MD, Kouros-Mehr H, Lu P, Werb Z: **Hormonal and local control of mammary branching morphogenesis**. *Differentiation; research in biological diversity* 2006, **74**(7):365-381.

20. Hinck L, Silberstein GB: **Key stages in mammary gland development: the mammary end bud as a motile organ**. *Breast cancer research : BCR* 2005, **7**(6):245-251.

21. Sternlicht MD: **Key stages in mammary gland development: the cues that regulate ductal branching morphogenesis**. *Breast cancer research : BCR* 2006, **8**(1):201.

22. Wiseman BS, Sternlicht MD, Lund LR, Alexander CM, Mott J, Bissell MJ, Soloway P, Itohara S, Werb Z: **Site-specific inductive and inhibitory activities of MMP-2 and MMP-3 orchestrate mammary gland branching morphogenesis**. *The Journal of cell biology* 2003, **162**(6):1123-1133.

23. Angeloni SV, Martin MB, Garcia-Morales P, Castro-Galache MD, Ferragut JA, Saceda M: **Regulation of estrogen receptor-alpha expression by the tumor suppressor gene p53 in MCF-7 cells**. *The Journal of endocrinology* 2004, **180**(3):497-504.

24. Cattoretti G, Rilke F, Andreola S, D'Amato L, Delia D: **P53 expression in breast cancer**. *International journal of cancer Journal international du cancer* 1988, **41**(2):178-183.

25. Caleffi M, Teague MW, Jensen RA, Vnencak-Jones CL, Dupont WD, Parl FF: **p53 gene mutations and steroid receptor status in breast cancer. Clinicopathologic correlations and prognostic assessment**. *Cancer* 1994, **73**(8):2147-2156.

26. Olayioye MA: **Update on HER-2 as a target for cancer therapy: Intracellular signaling pathways of ErbB2/HER-2 and family members**. *Breast Cancer Research* 2001, **3**(6):385-389.

27. Alluri PG, Speers C, Chinnaiyan AM: **Estrogen receptor mutations and their role in breast cancer progression**. *Breast Cancer Research* 2014, **16**(6):494.

28. García-Becerra R, Santos N, Díaz L, Camacho J: **Mechanisms of resistance to endocrine therapy in breast cancer: focus on signaling pathways, miRNAs and genetically based resistance**. *International journal of molecular sciences* 2012, **14**(1):108-145.

29. Boonyaratanakornkit V, Edwards P: **Receptor mechanisms mediating non-genomic actions of sex steroids**. In: *Seminars in reproductive medicine: 2007*: Thieme; 2007: 139.

30. Tan H, Zhong Y, Pan Z: **Autocrine regulation of cell proliferation by estrogen receptor-alpha in estrogen receptor-alpha-positive breast cancer cell lines**. *BMC cancer* 2009, **9**(1):31.
